# Anthroposophic medical therapy in chronic disease: a four-year prospective cohort study

**DOI:** 10.1186/1472-6882-7-10

**Published:** 2007-04-23

**Authors:** Harald J Hamre, Claudia M Witt, Anja Glockmann, Renatus Ziegler, Stefan N Willich, Helmut Kiene

**Affiliations:** 1 Institute for Applied Epistemology and Medical Methodology, Böcklerstr. 5, 79110 Freiburg, Germany; 2 Institute of Social Medicine, Epidemiology, and Health Economics, Charité University Medical Center, Campus Mitte, 10098 Berlin, Germany; 3 Society for Cancer Research, Kirschweg 9, 4144 Arlesheim, Switzerland

## Abstract

**Background:**

The short consultation length in primary care is a source of concern, and the wish for more consultation time is a common reason for patients to seek complementary medicine. Physicians practicing anthroposophic medicine have prolonged consultations with their patients, taking an extended history, addressing constitutional, psychosocial, and biographic aspect of patients' illness, and selecting optimal therapy. In Germany, health benefit programs have included the reimbursement of this additional physician time. The purpose of this study was to describe clinical outcomes in patients with chronic diseases treated by anthroposophic physicians after an initial prolonged consultation.

**Methods:**

In conjunction with a health benefit program in Germany, 233 outpatients aged 1–74 years, treated by 72 anthroposophic physicians after a consultation of at least 30 min participated in a prospective cohort study. Main outcomes were disease severity (Disease and Symptom Scores, physicians' and patients' assessment on numerical rating scales 0–10) and quality of life (adults: SF-36, children aged 8–16: KINDL, children 1–7: KITA). Disease Score was documented after 0, 6 and 12 months, other outcomes after 0, 3, 6, 12, 18, 24, and (Symptom Score and SF-36) 48 months.

**Results:**

Most common indications were mental disorders (17.6% of patients; primarily depression and fatigue), respiratory diseases (15.5%), and musculoskeletal diseases (11.6%). Median disease duration at baseline was 3.0 years (interquartile range 0.5–9.8 years). The consultation leading to study enrolment lasted 30–60 min in 51.5% (120/233) of patients and > 60 min in 48.5%. During the following year, patients had a median of 3.0 (interquartile range 1.0–7.0) prolonged consultations with their anthroposophic physicians, 86.1% (167/194) of patients used anthroposophic medication.

All outcomes except KITA Daily Life subscale and KINDL showed significant improvement between baseline and all subsequent follow-ups. Improvements from baseline to 12 months were: Disease Score from mean (standard deviation) 5.95 (1.74) to 2.31 (2.29) (p < 0.001), Symptom Score from 5.74 (1.81) to 3.04 (2.16) (p < 0.001), SF-36 Physical Component Summary from 44.01 (10.92) to 47.99 (10.43) (p < 0.001), SF-36 Mental Component Summary from 42.34 (11.98) to 46.84 (10.47) (p < 0.001), and KITA Psychosoma subscale from 62.23 (19.76) to 76.44 (13.62) (p = 0.001). All these improvements were maintained until the last follow-up. Improvements were similar in patients not using diagnosis-related adjunctive therapies within the first six study months.

**Conclusion:**

Patients treated by anthroposophic physicians after an initial prolonged consultation had long-term reduction of chronic disease symptoms and improvement of quality of life. Although the pre-post design of the present study does not allow for conclusions about comparative effectiveness, study findings suggest that physician-provided anthroposophic therapy may play a beneficial role in the long-term care of patients with chronic diseases.

## Background

The short consultation length in primary care (average 7½–15½ min in seven European countries [1,2]) is a long-standing source of concern [3,4]. Physicians who have short consultations give less lifestyle advice and have less satisfied patients, but it is not clear if the amount of time spent is the causative factor or a marker for other attributes [4]. In intervention studies to increase consultation length, the average duration was increased by only 1–3 minutes, patient satisfaction was not improved, and effects on patients' health were not evaluated [5].

Whereas sufficient time with the physician is a high priority for primary care patients [6], the wish for more consultation time is also a common reason for patients to seek complementary medicine [7]. Some complementary therapies are provided by primary care physicians, and these physicians have prolonged consultations, providing therapies such as acupuncture, homeopathy, or anthroposophic medicine (AM).

AM is a complementary system of medicine founded by Rudolf Steiner and Ita Wegman [8], provided by physicians and non-medical practitioners. AM acknowledges a spiritual-existential dimension in man which is assumed to interact with psychological and somatic levels in health and disease. AM therapy for chronic disease aims to counteract constitutional vulnerability, stimulate salutogenetic self-healing capacities, and strengthen patient autonomy [9-11]. This is sought to be achieved by counselling [10], by non-verbal artistic therapies using painting or clay [12,13], music [14] or speech exercises [15], by eurythmy movement exercises [16], by physical therapies [17,18], and by special medication. A key concept of AM medication therapy is typological correspondences between pathophysiological processes in man and formative forces working in minerals, plants and animals, reflecting a common evolution of man and nature [8-11]. These correspondences are used therapeutically in medications of mineral, botanical or zoological origin. The manufacturing of AM medication includes special pharmaceutical processes which are rarely used for non-AM medication: e.g. the production of metal mirrors by chemical vapour decomposition, and the processing of herbs by fermentation, toasting, carbonising, incineration or digestion (heat treatment at 37°C). AM medications can be prepared in concentrated form or in homoeopathic potencies, and are administered in various ways (oral, rectal, vaginal, conjunctival, nasal or percutaneous application, or by subcutaneous, intracutaneous or intravenous injection). Currently, more than 2000 different AM medications are on the market [19,20].

Certification as an AM physician requires a completed medical degree and a structured postgraduate AM training according to international criteria [21]. In Europe, more than 2000 AM physicians provide comprehensive AM therapy in inpatient and outpatient settings [21]. Worldwide, AM physicians work in 56 countries [22].

AM physicians have prolonged consultations with their patients. These consultations are used to take an extended history, to address constitutional, psychosocial, and biographic-existential aspect of patients' illness, to explore the patient's preparedness to engage in treatment, and to select optimal therapy for each patient [9,10,23]. The importance of addressing psychosocial and biographic aspects of illness is also recognised in conventional medicine [24], and in this respect AM practice has been studied as a possible model of integrated primary care [10].

In Germany, several health benefit programs ("Modellvorhaben") have included the reimbursement of additional time spent by physicians providing complementary therapies [25-27]. We here present a study of patients treated by AM physicians after an initial AM-related consultation of at least 30 minutes duration.

## Methods

### Study design and objective

This is a prospective four-year cohort study in a real-world medical setting. The study was part of a research project on the effectiveness and costs of AM therapies in outpatients with chronic disease (Anthroposophic Medicine Outcomes Study, AMOS) [26,28]. The AMOS project was initiated by a health insurance company in conjunction with a health benefit program. The primary research question of the present study was: Is physician-provided AM therapy after an initial prolonged AM-related consultation associated with clinically relevant improvement of symptoms? Further research questions concerned quality of life, use of adjunctive therapies and health services, adverse reactions, and therapy satisfaction.

### Setting, participants, and therapy

All physicians certified by the Physicians' Association for Anthroposophical Medicine in Germany and working in an office-based practice or outpatient clinic in Germany were invited to participate in the study. The participating physicians were instructed to enrol consecutive patients fulfilling eligibility criteria. Inclusion criteria were (1) outpatients aged 1–75 years, (2) an initial AM-related consultation ≥ 30 min with the study physician for any indication (main diagnosis). Exclusion criteria were previous AM-related consultation ≥30 min for the main diagnosis. Patients were treated according to the physicians' discretion. Therapies were classified as: physician-provided AM therapy (AMT: AM-related consultations with study physicians, AM medication), AM adjunctive therapy (AM art therapy, eurythmy therapy), non-AM adjunctive therapy (all other therapies).

### Clinical outcomes

• Disease severity was assessed on numerical rating scales [29] from 0 („not present“) to 10 („worst possible“): Disease Score (physician's global assessment of severity of main diagnosis, documented in patients enrolled up to 30 Sep 2000); Symptom Score (patients' assessment of one to six most relevant symptoms present at baseline, documented in patients enrolled after 1 Jan 1999).

• Quality of life was assessed with SF-36^® ^Physical and Mental Component Summary Measures, the eight SF-36 subscales, and the SF-36 Health Change item [30] for adults; with KINDL^® ^40-item version, Summary Score and four subscales [31] for children 8–16 years; and with KITA Psychosoma and Daily Life subscales [32] for children 1–7 years.

Disease Score was documented after 0, 6 and 12 months, other clinical outcomes after 0, 3, 6, 12, 18, 24, and (Symptom Score and SF-36) 48 months.

### Other outcomes

• Adjunctive therapy and health service use in the pre-study year was documented at study enrolment, use in the first study year was documented after six and 12 months, and use in the second study year was documented after 18 and 24 months. Items were: medication (additional documentation after three months), physician and dentist visits, paraclinical investigations, inpatient hospital and rehabilitation treatment, surgeries, physiotherapy, ergotherapy, psychotherapy, Heilpraktiker (non-medical practitioner) visits, and sick leave.

• Use of diagnosis-related non-AM adjunctive therapies within the first six study months was analysed in patients with a main diagnosis of mental, respiratory or musculoskeletal diseases, or headache syndromes. Diagnosis-related therapies were any of the following therapies, if used for at least one day per month: Mental diseases: psychotherapy (in children ergotherapy or play therapy), antiepileptic, psycholeptic, analeptic, and anti-addiction drugs (ATC-Index N03A, N05-06, N07B); Respiratory diseases: relevant drugs (H02, J01-02, J04-05, J07A, L03, R01, R03, R06-07) or surgery; Musculoskeletal diseases: immunosuppressive, musculoskeletal, analgesic and antidepressant drugs (L04, M01-05, M09, N02A-B, N06A), physiotherapy or relevant surgery; Headache disorders: analgesics, antimigraine drugs and antidepressants (C04AX01, C07AA05, C07AB02, C08CA06, C08DA01, N02, N03AG01, N06A, N07CA03).

• Therapy ratings were documented after six and 12 months: Patient rating of therapy outcome, patient satisfaction with therapy, therapy effectiveness rating by patient and physician.

• Adverse drug or therapy reactions were documented during the first 24 study months: cause, intensity (mild/moderate/severe = no/some/complete impairment of normal daily activities); Serious Adverse Events (physician documentation).

### Data collection

All data were documented with questionnaires sent in sealed envelopes to the study office. Physicians documented eligibility criteria; all other items were documented by patients (by caregivers of children < 17 years) unless otherwise stated. Patient responses were not made available to physicians. Physicians were compensated € 40 per included and fully documented patient, while patients received no compensation.

Data were entered twice by two different persons into Microsoft^® ^Access 97. The two datasets were compared and discrepancies resolved by checking with the original data.

### Quality assurance, adherence to regulations

The study was approved by the Ethics Committee of the Faculty of Medicine Charité, Humboldt University Berlin, and was conducted according to the Helsinki Declaration and the International Conference on Harmonisation Good Clinical Practice guidelines. Written informed consent was obtained from all patients before enrolment.

### Data analysis

Data analysis (SPSS^® ^13.0.1, StatXact^® ^5.0.3) was performed on all patients fulfilling eligibility criteria. For continuous data the Wilcoxon Signed-Rank test was used for paired samples and the Mann-Whitney U-test for independent samples; median differences with 95% confidence intervals (95%-CI) were estimated according to Hodges and Lehmann [33]. For binominal data McNemar test and Fisher's exact test were used. All tests were two-tailed. Significance criteria were p < 0.05 and 95%-CI not including 0. Pre-post effect sizes were calculated as Standardised Response Mean (= mean change score divided by the standard deviation of the change score) and classified as small (0.20–0.49), medium (0.50–0.79), and large (≥0.80) [34]. Unless otherwise stated, therapies and health services were analysed in patients enrolled after 1 Jan 1999 with at least three out of five follow-ups available; for each item and follow-up period, missing values were replaced by the group mean value. Clinical outcomes were analysed in patients with evaluable data for each follow-up, without replacement of missing values.

## Results

### Participating physicians

78 physicians screened patients. 72 physicians enrolled patients into the study; these physicians did not differ significantly from all certified AM physicians in Germany (n = 362) regarding gender (59.7% vs. 62.2% males), age (mean 45.4 vs. 47.5 years), number of years in practice (17.5 vs. 19.5), or the proportion of primary care physicians (80.6% vs. 85.0%).

### Patient recruitment and follow-up

**Figure 1:**
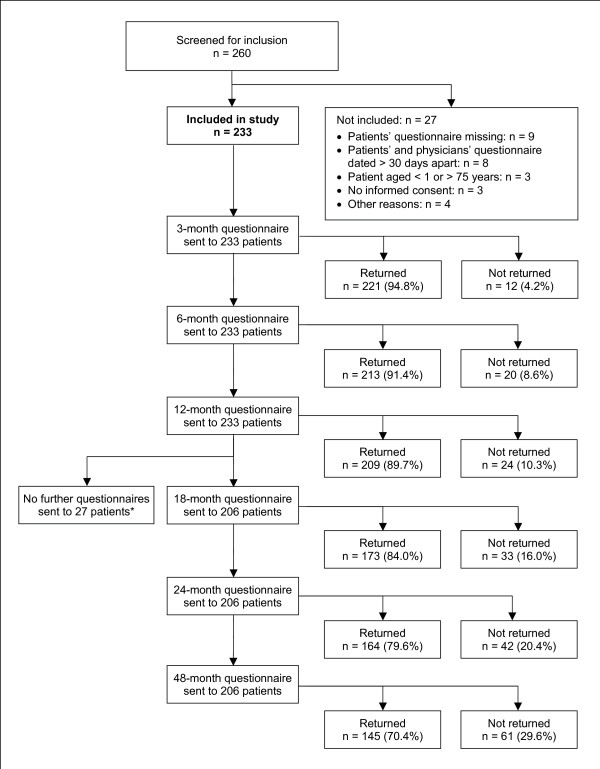
**Patient recruitment and follow-up**. *18-, 24-, and 48-month follow-up questionnaires were not sent to patients enrolled before 1 Jan 1999.

From 1 July 1998 to 31 March 2001, a total of 260 patients were screened for inclusion. 233 patients fulfilled all eligibility criteria and were included in the study (Figure 1). Of the 233 included patients, 13 patients were also included in a study of depression [35], and one patient was included in a study of low back pain [36]. The last patient follow-up ensued on 27 March 2005. Included and not included patients did not differ significantly regarding age, gender, diagnosis, disease duration, baseline Disease Score, or baseline Symptom Score.

73.8% (172/233) of patients were enrolled by general practitioners, 9.4% by paediatricians, 4.7% by internists, and 12.0% by other specialists. The physicians' setting was primary care practice (84.1% of patients, n = 196/233), referral practice (9.0%), and outpatient clinic (6.9%).

98.3% (229/233) of patients returned at least one follow-up questionnaire. The 12-month questionnaire was returned by 89.7% of patients; these patients did not differ significantly from non-respondents (10.3%) regarding age, gender, diagnosis, disease duration, baseline Disease Score, or baseline Symptom Score. Corresponding dropout analyses for the 24-month follow-up also showed no differences. The physician follow-up documentation was available for 84.5% (197/233) of patients after six months and for 81.7% after 12 months.

### Baseline characteristics

Most frequent main diagnoses, classified by ICD-10 (International Classification of Diseases, Tenth Edition), were F00-F99 Mental Disorders (17.6%, 41/233 patients) J00-J99 Respiratory Diseases (15.5%), M00-M99 Musculoskeletal Diseases (11.6%), G00-G99 Nervous System Diseases (9.9%), and L00-L99 Skin and Subcutaneous Tissue Diseases (9.0%). Most frequent single diagnoses were depression (6.9%, 16/233 patients), headache/migraine (5.6%), recurrent infections (3.9%), fatigue (3.4%), asthma (3.4%), and atopic dermatitis (3.4%).

Median disease duration was 3.0 years (interquartile range (IQR) 0.5–9.8 years, mean 34.9 years). The patients had median 1.0 (IQR 1.0–2.0) comorbid diseases. Most common comorbid diseases, classified by ICD-10, were M00-M99 Musculoskeletal Diseases (17.0%, 56 of 329 diagnoses), J00-J99 Respiratory Diseases (9.7%), and F00-F99 Mental Disorders (9.4%).

The patients were recruited from 15 of 16 German federal states. Median age was 38.0 years (IQR 23.0–47.0, range 1.0–74.0 years, mean 34.9 years). Compared to the German population, socio-demographic items were more favourable for education, occupation, income, alcohol, smoking in females, overweight, sport, and severe disability status; items were similar for unemployment, living alone, smoking in males, and underweight; and were less favourable for work disability pension and sick-leave (Table 1).

**Table 1:** Socio-demographic data

		**Study patients**		**German primary care patients**	
**Items**		**N**	**Percent**	**Percent**	**Source**

Female gender		169/233	73%	53%	[45]
Age groups	0–19 years	54/233	23%	14%	[45]
	20–39 years	73/233	31%	27%	[45]
	40–59 years	87/233	37%	27%	[45]
	60–75 years	19/233	8%	21%	[45]

		**Adult study patients enrolled after 1. Jan 1999**		**German population**	

"Fachhochschule" or university entrance qualification		69/157	44%	19%	[46]
University degree		32/156	21%	6%	[46]
Wage earners		7/157	4%	18%	[46]
Unemployed during last 12 months	Economically active patients	7/91	8%	10%	[46]
Living alone		25/154	16%	21%	[46]
Net family income < 900 € per month		15/136	11%	16%	[46]
Alcohol use daily (EYT) vs. almost daily (Germany)	Male	4/32	13%	28%	[47]
	Female	3/125	2%	11%	
Regular smoking	Male	14/32	44%	37%	[48]
	Female	18/124	15%	28%	
Sports activity ≥ 1 hour weekly	Age 25–69	71/141	50%	39%	[49]
Body mass index < 18.5 (low)	Male	0/32	0%	1%	[50]
	Female	6/124	5%	4%	
Body mass index ≥ 25 (overweight)	Male	9/32	28%	56%	[50]
	Female	32/124	26%	39%	
Permanent work disability pension		9/157	6%	3%	[51]
Severe disability status		8/157	5%	12%	[52]
Sick leave days in the last 12 months, mean (SD)	Economically active patients	22.4 (44.8) days		17.0 days	[53]

### Therapies

The duration of the initial AM-related consultation leading to study enrolment was 30–45 min in 35.2% (82/233) of patients, 45–60 min in 16.3%, and >60 min in 48.5%. During the following year, the patients had median 3.0 (IQR 1.0–7.0) further AM-related consultations with their study physician, thereof median 1.0 (IQR 0.0–3.0) consultation ≥60 min and 0.5 (IQR 0.0–2.0) consultation 45–60 min. AM medication was used by 71.1% (138/194) of patients during the first three months after study enrolment, by 83.0% during the first six months, and by 86.1% during the first 12 months (analysed in patients with complete follow-up data after 3, 6, and 12 months; see also Table 2). During the first six study months 3.8% (8/211) of evaluable patients had AM art therapy, and 14.2% (30/211) had eurythmy therapy.

**Table 2:** AM medication, non-AM adjunctive therapies, health service use, and sick leave days

**Item**	**Pre-study year**	**0–12 months**			**12–24 months**		
	**Mean (SD)**	**Mean (SD)**	**Median difference (95%-CI) from pre-study year**	**P value**	**Mean (SD)**	**Median difference (95%-CI) from pre-study year**	**P value**

AM medicines per day	0.29 (0.68)	0.78 80.92)	0.47 (0.36 to 0.59)	p < 0.001	0.47 (0.85)	0.17 (0.09 to 0.26)	p < 0.001
Non-AM medicines per day	0.69 (1.10)	0.82 (1.01)	0.12 (0.04 to 0.22)	p = 0.005	0.68 (0.90)	0.04 (-0.05 to 0.15)	p = 0.447
Physician and dentist visits	15.23 (15.27)	16.64 (13.53)	1.69 (0.19 to 2.99)	p = 0.006	14.84 (14.35)	-0.78 (-2.43 to 1.00)	p = 0.698
Paraclinical investigations	5.16 (5.91)	5.85 (7.52)	0.00 (-0.50 to 1.00)	p = 0.665	4.34 (5.32)	-0.98 (-1.50 to -0.08)	p = 0.011
Hospital days	4.68 (16.25)	1.77 (5.82)	-2.50 (-8.00 to 1.00)	p = 0.123	3.04 (19.92)	-2.50 (-5.50 to 0.00)	p = 0.046
Rehabilitation days	0.89 (5.05)	1.74 (6.75)	7.00 (0.00 to 14.48)	p = 0.041	0.67 (3.43)	-0.62 (-0.90 to -0.56)	p = 0.008
Surgeries	0.21 (0.51)	0.17 (0.49)	0.00 (-0.50 to 0.00)	p = 0.342	0.14 (0.40)	-0.34 (-0.46 to 0.07)	p = 0.494
Physiotherapy and ergotherapy sessions	9.25 (25.98)	11.28 (34.02)	1.50 (-2.50 to 6.00)	p = 0.541	9.88 (25.95)	0.22 (-3.59 to 5.10)	p = 0.885
Psychotherapy sessions	2.50 (8.04)	3.12 (9.06)	3.50 (0.50 to 7.42)	p = 0.023	3.58 (9.88)	2.78 (1.72 to 6.52)	p = 0.007
Sick leave days*	22.42 (44.79)	27.61 (71.91)	1.24 (-7.50 to 9.00)	p = 0.748	26.39 (62.96)	0.50 (-7.00 to 8.18)	p = 0.823
Patients with Heilpraktiker visit (n + %)**	21/143 (14.7%)	18/143 (12.6%)		p = 0.571	22/143 (15.4%)		p = 1.000

Patients enrolled after 1 Jan 1999 with at least 3 of 5 follow-ups (n = 182). *Patients engaged in economic activity (n = 80). **Patients with complete data for all time periods.

Non-AM adjunctive therapies, health services, and sick leave are listed in Table 2, together with AM medication. Comparing the pre-study year to the first and second study year, respectively, the only consistent changes over both years were increases in AM medication use and psychotherapy. In the first study year the number of physician and dentist visits increased by median 1.7 (average 1.4) visits; notably this number (average 16.6 in first year) includes AM-related consultations with the study physicians (average 5.2).

Use of diagnosis-related non-AM adjunctive therapies (see Methods) within the first six study months was analysed in patients with a main diagnosis of mental, respiratory or musculoskeletal diseases, or headache syndromes (n = 117). Out of 103 evaluable patients, 63% (n = 65) had no diagnosis-related adjunctive therapy.

### Clinical outcomes

**Figure 2:**
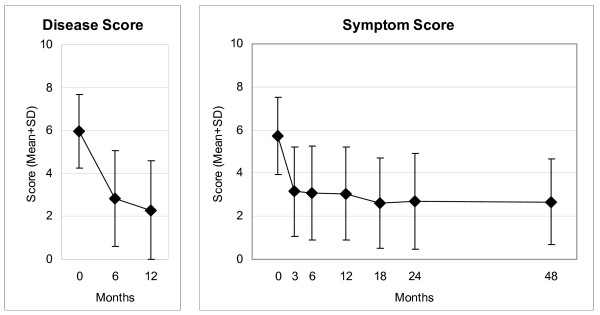
**Disease and Symptom Scores**. Disease Score: physicians' assessment, Symptom Score: patients' assessment. Range 0 "not present", 10 "worst possible".

**Figure 3:**
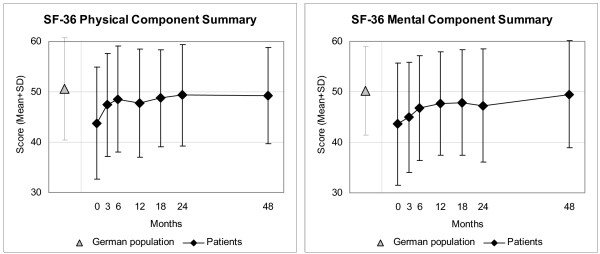
**SF-36 Physical and Mental Component Summary Measures**. Higher scores indicate better health. Adult patients and German population (standardised for age and gender) [30]

**Figure 4:**
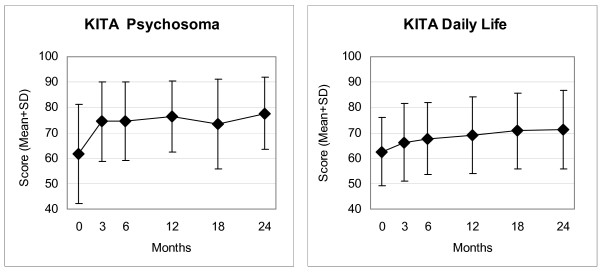
**KITA Psychosoma and Daily Life subscales**. Range 0–100, higher scores indicate better health. Children aged 1–7 years.

**Figure 5:**
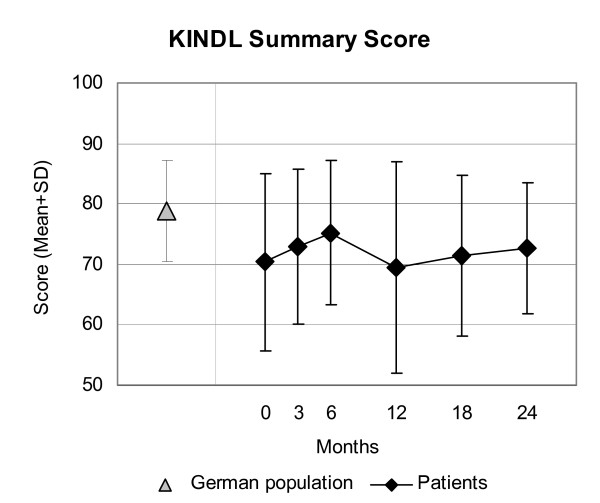
**KINDL Summary Score**. Range 0–100, higher scores indicate better health. Children aged 8–16 years and German population sample (9–12 years) [31].

Disease Score (Figure 2), Symptom Score (Figure 2), all eleven SF-36 scores (adults, Figure 3), and the KITA Psychosoma subscale (children aged 1–7, Figure 4) improved significantly between baseline and nearly all subsequent follow-ups (75 significant and four non-significant improvements in 79 pre-post comparisons). For all these 14 outcomes, the most pronounced improvement occurred during the first six months. After 12-months, Disease and Symptom Scores were improved from baseline in 88.4% and 83.2% of patients, respectively (Table 3); an improvement of ≥50% of baseline scores was observed in 69.0% (107/155 evaluable patients) and 48.4% (89/184), respectively. Disease and Symptom Scores improved similarly in adults and in children. Effect sizes for the 0–12 month comparison were large for Disease and Symptom Scores (1.52 and 1.05) and small-to-medium (range 0.33–0.71) for the SF-36 scores and KITA Psychosoma (Table 3). All these improvements were maintained until the last follow-up. KINDL scores (children aged 8–16, Figure 5) and the KITA Daily Life subscale (children aged 1–7, Figure 4) did not change significantly during the study (exceptions: two significant improvements in 30 pre-post comparisons).

**Table 3:** Clinical outcomes 0–12 months

**Item**	**N**	**0 months**	**12 months**	**0 months vs. 12 months**			
		**Mean (SD)**	**Mean (SD)**	**P-value**	Median difference (95%-CI)*	**Improved**	**SRM**

Disease Score (0–10)	155	5.95 (1.74)	2.31 (2.29)	p < 0.001	4.00 (3.50 to 4.50)	88%	1.52
Symptom Score (0–10)	184	5.74 (1.81)	3.04 (2.16)	p < 0.001	2.97 (2.50 to 3.25)	83%	1.05
SF-36 scales (0–100)							
-Physical Function	161	77.09 (24.07)	84.41 (20.69)	p < 0.001	7.50 (5.00 to 10.00)	60%	0.37
-Role Physical	157	55.47 (38.39)	72.29 (36.64)	p < 0.001	25.00 (12.50 to 37.50)	46%	0.42
-Role-Emotional	157	68.15 (37.43)	81.63 (31.98)	p < 0.001	33.33 (16.67 to 33.34)	39%	0.33
-Social Functioning	161	67.93 (25.07)	77.10 (23.06)	p < 0.001	12.50 (6.25 to 18.75)	52%	0.34
-Mental Health	160	58.74 (18.25)	66.10 (18.34)	p < 0.001	8.00 (4.00 to 10.00)	63%	0.43
-Bodily Pain	161	59.89 (27.62)	71.27 (26.76)	p < 0.001	15.50 (9.50 to 21.00)	54%	0.38
-Vitality	160	42.53 (18.34)	52.84 (18.49)	p < 0.001	12.50 (10.00 to 17.50)	60%	0.53
-General Health	161	53.10 (19.30)	61.01 (19.48)	p < 0.001	8.50 (5.00 to 11.00)	63%	0.45
SF-36 Health Change (1–5**)	159	3.27 (1.08)	2.14 (1.02)	p < 0.001	1.50 (1.00 to 2.00)	65%	0.71
SF-36 Physical Component	154	44.01 (10.92)	47.99 (10.43)	p < 0.001	3.97 (2.71 to 5.30)	73%	0.42
SF-36 Mental Component	154	42.34 (11.98)	46.84 (10.47)	p < 0.001	3.98 (2.30 to 5.71)	64%	0.40
KINDL subscales (0–100)							
-Psychic	16	69.75 (19.91)	69.74 (21.57)	p = 0.782	1.13 (-4.55 to 5.68)	56%	0.00
-Somatic	16	71.18 (17.21)	71.74 (19.88)	p = 0.520	2.78 (-4.16 to 8.33)	63%	0.03
-Social	16	74.30 (14.89)	69.73 (18.78)	p = 0.292	-4.16 (-9.72 to 3.65)	31%	-0.26
-Function	15	67.42 (12.08)	69.18 (14.45)	p = 0.367	2.04 (-3.41 to 6.82)	67%	0.20
KINDL Summary Score (0–100)	16	70.39 (17.67)	69.77 (17.91)	p = 0.889	0.31 (-4.02 to 4.93)	50%	-0.06
KITA subscales (0–100)							
-Psychosoma	31	62.23 (19.76)	76.44 (13.62)	p = 0.001	13.54 (4.17 to 21.87)	77%	0.62
-Daily Life	29	63.22 (12.40)	67.96 (15.43)	p = 0.038	6.25 (0.00 to 12.50)	55%	0.28

*Positive differences indicate improvement. Improved: Percentage of patients improved from baseline. **1 = "much better now than one year ago", 5 = "much worse now than one year ago". SRM: Standardised Response Mean effect size (small: 0.20–0.49, medium: 0.50–0.79, large: ≥ 0.80).

We performed three post-hoc sensitivity analyses of 0–12 month Disease and Symptom Score outcomes. The first sensitivity analysis concerned dropout bias. The main analysis had comprised all patients with evaluable data at baseline and 12-month follow-up. In the first sensitivity analysis, missing values after 12 months were replaced with the last value carried forward, reducing the average 0–12 month Disease and Symptom Score improvements by 15% (3.65→3.12 points) and 5% (2.69→2.56 points), respectively. The second analysis concerned the effect of AM adjunctive therapies: The sample was restricted to patients using neither eurythmy nor art therapy in the first six study months, whereby the average improvement was increased by 4% for Disease Score (3.65→3.78) and was virtually unchanged for Symptom Score (2.69→2.68 points). The third analysis concerned the effects of relevant non-AM adjunctive therapies, and was performed on patients with a main diagnosis of mental, respiratory or musculoskeletal diseases or headache syndromes. Restricting this sample to patients not using diagnosis-related adjunctive therapies during the first six study months (see Methods), the average improvement was increased by 4% for Disease Score (3.84→3.98) and was virtually unchanged for Symptom Score (2.77→2.76 points).

### Other outcomes

At six-month follow-up, patients' average therapy outcome rating (numeric scale from 0 "no help at all" to 10 "helped very well") was 7.21 (SD 2.64); patient satisfaction with therapy (from 0 "very dissatisfied" to 10 "very satisfied") was 7.81 (2.45). Patients' therapy effectiveness rating was positive ("very effective" or "effective") in 75.5% (157/208) of patients, and negative ("less effective", "ineffective" or "not evaluable") in 24.5%. Physicians' effectiveness rating was positive in 80.9% (157/194) and negative in 19.1%. Ratings of therapy outcome, satisfaction and effectiveness did not differ significantly between adults (patient rating) and children (proxy rating by caregivers), or between six- and 12-month follow-ups.

Adverse reactions to AM medication were reported in five (2.5%) of 197 patients using AM medication at least once during 24-month follow-up (causal relationship confirmed in four patients, not confirmed in one patient [20]). The intensity of these reported reactions was mild in one patient and moderate in four patients. Three (1.5%) patients stopped AM medication use because of suspected adverse reactions: Chamomilla/Malachit comp. (burning eyes), Conchae D6 (increased anxiety), and Oxalis 30% ointment (allergic exanthema). Adverse drug reactions from non-AM medication were reported in 13.5% (28/207) of users; medication was stopped in seven patients. No adverse reactions to adjunctive AM eurythmy or art therapies occurred but six patients had adverse reactions to non-AM non-drug therapies (surgery: n = 2, dental treatment: n = 2, acupuncture: n = 2).

Three patients had Serious Adverse Events. One patient died from gastric carcinoma and two were acutely hospitalised for thrombosis of lower extremity and Henoch-Schönlein purpura, respectively. None of these SAE Serious Adverse Events were related to any therapy or medication.

## Discussion

This prospective cohort study is the first study of comprehensive AMT for chronic disease performed in primary care. We aimed to obtain information on AMT under routine conditions in Germany and studied clinical outcomes in outpatients starting AMT for chronic diseases after an initial AM-related consultation of at least 30 min. The study was conducted in conjunction with a health insurance program reimbursing AM-related physician consultations regardless of diagnosis. For this reason, and because the range and frequency of indications for AMT in primary care was largely unknown prior to the study, we included patients of all ages with all diagnoses. Most frequent indications were mental, respiratory, and musculoskeletal disorders. Following AMT, significant improvements of disease symptoms and quality of life were observed. The largest improvements (large effect sizes, half of patients improved by at least 50% of their baseline scores) were observed for the items which directly measure the conditions treated with AMT, i.e. Disease and Symptom Scores. The improvements were maintained during the four-year follow-up and were not accompanied by an increase of adjunctive therapies, except for a small increase in psychotherapy use.

### Strengths and limitations

Strengths of this study include a long follow-up period, high follow-up rates, and the participation of 20% of all AM-certified physicians in Germany. The participating physicians resembled all eligible physicians with respect to socio-demographic characteristics, and the included patients resembled not included, screened patients regarding baseline characteristics. These features suggest that the study to a high degree mirrors contemporary AMT practice. Moreover, in the present early phase of AMT evaluation, the inclusion of all diagnoses is an advantage, offering a comprehensive picture of AMT practice. On the other hand, it was not feasible to have disease-specific outcomes for all diagnoses included. Nonetheless, the larger AMOS project, of which this study is part, included disease-specific outcomes for major disease groups [35,36].

Since the study had a long recruitment period, the participating physicians were not able to screen and include all their eligible patients (patients starting AMT after prolonged AM-related consultations). The degree of selection is not known for this part of the AMOS project, but for other parts (patients referred to AM therapies) it was estimated that physicians enrolled every fourth eligible patient [35]. This selection could bias results if physicians were able to predict therapy response and if they preferentially screened and enrolled such patients for whom they expected a particularly favourable outcome. In this case one would expect the degree of selection (= the proportion of eligible vs. enrolled patients) to correlate positively with clinical outcomes. That was not the case, the correlation was almost zero (-0.04). This analysis [35] does not suggest that physicians' screening of eligible patients was affected by selection bias.

A limitation of the study is the absence of a comparison group receiving another treatment or no therapy. Accordingly, for the observed improvements one has to consider several other causes apart from AMT. Non-AM adjunctive therapies cannot explain the improvement, since there was comparable improvement in patients not using such therapies (analysed in patients with mental, respiratory or musculoskeletal disease or headache syndromes, together comprising 50% of the study sample); the same applies to adjunctive AM art and eurythmy therapy. Dropout bias could explain up to 15% of the 0–12-month improvement of Disease Score but only 4% of the corresponding Symptom Score improvement. Natural recovery and regression to the mean, which could also bias results, will be addressed in a separate analysis (Hamre et al, submitted for publication). Other possible confounders are observation bias and psychological factors like patient expectations. Since, however, AMT was evaluated as a therapy package including physician-patient consultations, the question of specific therapy effects vs. non-specific effects (placebo effects, context effects, patient expectations etc.) was not an issue of the present analysis.

### Study implications

This study confirms previous studies of the characteristics of AM users [10,37-40]: Patients are predominantly middle-aged women or children, education and occupation levels are higher than average, and typical indications are mental, respiratory, and musculoskeletal disorders. Previous studies of comprehensive AMT for chronic disease have been conducted in inpatient settings (nine studies) and outpatient clinics (four studies with range 18–54 patients [41-44]). The latter four studies showed: improvement of symptoms and functional capacity and decreased local and systemic inflammatory activity in patients with inflammatory rheumatic disorders treated largely without conventional antirheumatic medication [41], successful epilepsy therapy without conventional anticonvulsive drugs [42], high response rates in hepatitis C patients treated without interferon [43], and decreased asthma symptoms in children treated without glucocorticoids [44]. In accordance with these findings from secondary care, our predominantly primary care study demonstrated long-standing improvements of disease symptoms and quality of life in patients with mental, respiratory, and musculoskeletal diseases and other chronic conditions.

In German general practice, a physician will spend an average of 7½ minutes with each patient [1]. In this study, physicians spent at least 30 minutes and provided AMT (AM-related consultations and AM medication). Study results show that these interventions can be associated with favourable clinical outcomes.

## Conclusion

In this study, patients treated by AM physicians after an initial prolonged consultation had long-term reduction of chronic disease symptoms and improvement of quality of life. Although the pre-post design of the present study does not allow for conclusions about comparative effectiveness", study findings suggest that physician-provided AM therapy may play a beneficial role in the long-term care of patients with chronic diseases.

## List of abbreviations

AM: anthroposophic medicine, AMOS: Anthroposophic Medicine Outcomes Study, AMT: AM-related consultations with study physician + AM medication, IQR: interquartile range.

### 

## Competing interests

Within the last five years HJH has received restricted research grants from the pharmaceutical companies Weleda and Wala, who produce AM medications. Otherwise all authors declare that they have no competing interests.

## Authors' contributions

HJH, CMW, SNW, and HK contributed to study design. HJH, AG, and HK contributed to data collection. HJH, RZ, and HK wrote the analysis plan, HJH and AG analysed data. HJH was principal author of the paper, had full access to all data, and is guarantor. All authors contributed to manuscript drafting and revision and approved the final manuscript.

## Pre-publication history

The pre-publication history for this paper can be accessed here:


